# Search for Novel Biomarkers to Predict Cytochrome P450 2C19 Activity Using Untargeted Metabolomics of Human Plasma

**DOI:** 10.3390/metabo16090670

**Published:** 2026-09-11

**Authors:** Ayako Oda, Yosuke Suzuki, Teruhide Koyama, Jun Negami, Sakura Suzuki, Koudai Iino, Nao Yamagishi, Natsuki Kamio, Etsuko Ozaki, Yasuyuki Yamamoto, Masahiro Nakatochi, Yukihide Momozawa, Ryota Tanaka, Hiroyuki Ono, Takahiro Sumimoto, Ryosuke Tatsuta, Hiroki Itoh, Naoyuki Takashima, Keitaro Matsuo, Keiko Ohno

**Affiliations:** 1 Department of Medication Use Analysis and Clinical Research, Meiji Pharmaceutical University, 2-522-1 Noshio, Kiyose 204-8588, Tokyo, Japan; 2 Department of Epidemiology for Community Health and Medicine, Kyoto Prefectural University of Medicine, 465 Kajii-cho, Kawaramachi-Hirokoji, Kamigyo-ku, Kyoto 602-8566, Japan; 3 Faculty of Regional Creation, Nara Prefectural University, 10 Funabashi-cho, Nara City 630-8258, Nara, Japan; 4 Department of Nursing Faculty of Health and Medical Sciences, Kyoto University of Advanced Science, 18 Gotanda-cho, Yamanouchi, Ukyo-ku, Kyoto 615-8577, Japan; 5 Public Health Informatics Unit, Department of Integrated Health Sciences, Nagoya University Graduate School of Medicine, 65 Tsurumai-cho, Showa-ku, Nagoya 466-8550, Aichi, Japan; 6 Laboratory for Genotyping Development, RIKEN Center for Integrative Medical Sciences, 1-7-22 Suehiro-cho, Tsurumi-ku, Yokohama 230-0045, Kanagawa, Japan; 7 Department of Clinical Pharmacy, Oita University Hospital, 1-1 Idaigaoka, Hasama-machi, Yufu 879-5593, Oita, Japanitoh@oita-u.ac.jp (H.I.); 8 Division of Cancer Epidemiology and Prevention, Aichi Cancer Center, 1-1 Kanokoden, Chigusa-ku, Nagoya 464-8681, Aichi, Japan; 9 Department of Cancer Epidemiology, Nagoya University Graduate School of Medicine, 65 Tsurumai-cho, Showa-ku, Nagoya 466-8550, Aichi, Japan

**Keywords:** biomarker, cytochrome P450 2C19, gene polymorphism, untargeted metabolomics, ultra-performance liquid chromatography coupled to quadrupole time-of-flight mass spectrometry

## Abstract

**Background/Objectives**: Cytochrome P450(CYP)2C19 activity varies widely among individuals. As genetic factors, CYP2C19*2 and CYP2C19*3 alleles reduce CYP2C19 activity, while the CYP2C19*17 allele increases CYP2C19 activity. However, environmental and physiological factors can also influence individual CYP2C19 activity. In this study, we searched for novel endogenous biomarkers for CYP2C19 activity using CYP2C19 gene polymorphism data combined with results of untargeted metabolomic analysis. **Methods**: 431 general adults analyzed in the Kyoto J-MICC Study and 255 patients who visited Oita University Hospital were studied. Plasma samples were pretreated by solid-phase and liquid-liquid extraction and subjected to untargeted metabolomic analysis using ultra-performance liquid chromatography coupled to quadrupole time-of-flight mass spectrometry. Based on CYP2C19 gene polymorphism data, participants were classified into extensive metabolizers (EM), intermediate metabolizers (IM), and poor metabolizers (PM). Compounds showing significant differences in abundance among the three groups were considered candidate compounds for predicting CYP2C19 activity. The predictive performance of candidate compounds for CYP2C19 PM status was evaluated using covariate-adjusted receiver operating characteristic (ROC) analysis. **Results**: The normalized abundance of compounds with *m*/*z* 160.1342, 303.2319 (a fatty acyl or prenol lipid), 314.2309, 449.3238, 653.3021, 792.5744, and 811.5988 (a glycerophospholipid or sphingolipid), and 902.5404 (a fatty acyl) differed significantly among CYP2C19 EM, IM, and PM groups (*p* < 0.05), and these eight compounds were considered candidate compounds. Covariate-adjusted ROC analysis showed that none of the candidate compounds significantly improved the discrimination of CYP2C19 PM status. **Conclusions**: Untargeted metabolomics combined with CYP2C19 gene polymorphism data yielded eight compounds associated with CYP2C19 phenotype. Further studies are needed to evaluate the usefulness of these compounds as biomarkers of CYP2C19 activity.

## 1. Introduction

Cytochrome P450(CYP)2C19 is involved in the metabolism of many drugs used routinely in clinical practice, including omeprazole [1], clopidogrel [2], and abrocitinib [3]. CYP2C19 shows gene polymorphisms, with CYP2C19 c.681G>A (rs4244285, CYP2C19*2) causing non-functional CYP2C19 protein [4] and CYP2C19 c.636G>A (rs4986893, CYP2C19*3) producing inactive CYP2C19 protein [5]. On the other hand, CYP2C19 c.−3402C>T (rs11188072) and CYP2C19 c.−806C>T (rs12248560) show complete linkage [6,7], and the gene variant containing c.−3402T and c.−806T, which is designated CYP2C19*17, increases CYP2C19 activity [6]. Based on the combination of inherited alleles, CYP2C19 phenotypes can be classified into ultrarapid metabolizer (e.g., CYP2C19*17/*17), rapid metabolizer (e.g., CYP2C19*1/*17), normal metabolizer (e.g., CYP2C19*1/*1), intermediate metabolizer (e.g., CYP2C19*1/*2, CYP2C19*1/*3, CYP2C19*2/*17, CYP2C19*3/*17), and poor metabolizer (e.g., CYP2C19*2/*2, CYP2C19*3/*3, CYP2C19*2/*3) [8]. Although CYP2C19 genotyping is useful for predicting genetically determined enzyme activity, it does not capture non-genetic factors such as environmental factors, including co-administered drugs, and physiological factors, including inflammation, liver disease, and renal disease [9,10,11]. Consequently, genotype-based prediction may not always reflect the current metabolic phenotype of an individual. Therefore, evaluation metrics for assessing CYP2C19 activity, which consider both non-genetic and genetic factors, are required.

Proton pump inhibitors such as omeprazole are recommended by the European Medicines Agency and the US Food and Drug Administration as indices of CYP2C19 activity in clinical drug-drug interaction studies [12,13]. Although microdosing of omeprazole is recommended for determining CYP2C19 activity to avoid therapeutic or adverse effects [14,15,16], administration of probe drugs can cause allergic reactions. To circumvent this problem, endogenous biomarkers in biological samples are used in quantitative assessment of drug-metabolizing enzyme activities in individuals [17]. However, no useful endogenous biomarkers that specifically indicate CYP2C19 activity have been reported. The discovery of novel endogenous biomarkers for predicting CYP2C19 activity could enable assessment of individual CYP2C19 activity without the need for probe drug administration, thereby contributing to optimal use of CYP2C19 substrates.

Metabolomics is the comprehensive and simultaneous profiling of metabolites and reflects changes in metabolites resulting from genetic, environmental, physiological, and other factors [18]. Metabolomics is used to identify novel biomarkers that contribute to the characterization of individual variations in disease and treatment response, drug discovery and development, and management of disease progression [18,19]. Recently, some novel biomarkers that predict drug-metabolizing enzyme activities have been identified using an untargeted metabolomics approach. Solanidine and metabolites of solanidine in human plasma or urine [20,21], and five ω- or (ω-1)-hydroxylated medium-chain acylcarnitines in human urine [22] have been proposed to be novel biomarkers of CYP2D6 and CYP3A4, respectively. Thus, utilization of an untargeted metabolomics approach may contribute to the discovery of novel endogenous biomarkers for CYP2C19 activity, including undiscovered metabolites.

With this background, this study aimed to search for novel endogenous biomarkers in human plasma for predicting CYP2C19 activity. We obtained CYP2C19 gene polymorphism data from Japanese general adult and patient cohorts, and categorized them into CYP2C19 extensive metabolizers (EM), intermediate metabolizers (IM), or poor metabolizers (PM) according to CYP2C19 gene polymorphism. Metabolic profiles of all the subjects were constructed based on the results of untargeted metabolome analysis. We considered compounds showing significant differences in abundance between the three groups to be candidate compounds for predicting CYP2C19 activity. In addition, we evaluated the predictive performance of the candidate compounds using covariate-adjusted receiver operating characteristic (ROC) analysis.

## 2. Materials and Methods

### 2.1. Materials

Acetonitrile, formic acid, isopropanol, and methanol of liquid chromatography-mass spectrometry grade, and 28% ammonium hydroxide, ammonium formate, chloroform, and phosphoric acid of the highest analytical grade were purchased from FUJIFILM Wako Pure Chemical Corporation (Osaka, Japan). Ultra-pure water was obtained from the Synergy^®^ UV ultra-pure water system (Merck KGaA, Darmstadt, Germany). Ninety-six-well solid-phase extraction (SPE) plate (Oasis^®^ MAX μElution Plate and PRiME MCX μElution Plate), 96-well collection plate, and adhesive plate seal were purchased from Waters (Milford, MA, USA).

### 2.2. Participants

In this study, we utilized the database of the Japan Multi-Institutional Collaborative Cohort Study, containing cross-sectional data of the Japanese general adult population, and selected the data obtained in the Kyoto area (Kyoto J-MICC Study) [23,24,25]. The participants in the Kyoto J-MICC study were aged 35–69 years and consisted of community inhabitants and health check-up examinees. All participants signed written informed consent, and the study protocol was approved by the ethics committee of the Kyoto Prefectural University of Medicine (RBMR-E-267-10). From the Kyoto J-MICC database, we randomly selected data from 448 general adults who underwent health examinations at Kyoto Prefectural University of Medicine. The final cohort for analysis comprised 431 participants with body mass index (BMI) below 30 kg/m^2^, alanine aminotransferase (ALT) below 100 IU/L, total bilirubin below 2.0 mg/dL, and estimated glomerular filtration rate (eGFR) above 60 mL/min/1.73 m^2^. Seventeen individuals were excluded due to BMI 30 kg/m^2^ and over, total bilirubin 2.0 mg/dL and over, or lack of CYP2C19 gene polymorphism data. The following participant characteristics and laboratory data were collected from health examination records: sex, age, body weight, height, ALT, total bilirubin, and serum creatinine. Venous blood samples were collected in the morning under fasting conditions. All plasma samples were stored at −80 °C until untargeted metabolite analysis. This study is a secondary use of samples and data obtained from the Kyoto J-MICC Study. The requirement for written informed consent was waived by the ethics committee because the study involved secondary analysis of existing, anonymized samples and data. An opt-out procedure was implemented, and information about the study was publicly disclosed, allowing participants the opportunity to refuse participation.

We also analyzed 255 patients who visited Oita University Hospital and met the following selection criteria: over 18 years of age; not using therapeutic drugs that strongly or moderately induce or inhibit CYP2C19, such as phenytoin, fluconazole, and voriconazole; BMI below 30 kg/m^2^; ALT below 100 IU/L; and total bilirubin below 2.0 mg/dL. Venous blood samples were collected into a tube containing EDTA-3K anticoagulant in the morning under fasting conditions. Four hundred μL of whole blood was aliquoted for gene polymorphism measurement and stored at −40 °C until analysis. The remaining blood sample was centrifuged at 2330 *g* for 5 min at 4 °C, and plasma was transferred into a transparent polypropylene tube and stored at −80 °C until analysis. Sex, age, body weight, height, ALT, total bilirubin, serum creatinine, and underlying disease were extracted from electronic medical records. This study is a secondary use of samples and data obtained from a previous study (approval number: P-13-14), in which the scientific purpose and procedures were explained to each patient, and written informed consent was obtained. For the present study, the requirement for written informed consent was waived by the ethics committee because the study involved secondary analysis of existing, anonymized samples and data. An opt-out procedure was implemented, and information about the study was publicly disclosed, allowing participants the opportunity to refuse participation.

BMI was calculated as body weight (kg) divided by the square of height (m^2^). Estimated GFR was calculated using the Japanese equation incorporating serum creatinine, age, and sex [26].

This study was conducted in compliance with the Declaration of Helsinki and was approved by the ethics committees of Meiji Pharmaceutical University (approval numbers: 202230 and 202410, approval date: 20 October 2022 and 1 July 2024), Kyoto Prefectural University of Medicine (approval number: ERB-G-140, approval date: 26 December 2022), and Oita University Hospital (approval number: 2812-D29, approval date: 23 May 2024).

### 2.3. Genotyping Procedures for CYP2C19 and CYP2C9

For the general adult population, a total of 14,539 individuals from 12 regions in Japan recruited in the J-MICC study were genotyped at RIKEN Center for Integrative Medicine using the Human OmniExpressExome-8 version v1.2 BeadChip array (Illumina Inc., San Diego, CA, USA) [27]. Quality control filtering of samples and single nucleotide polymorphisms (SNPs) was conducted as reported previously [27]. Quality control filtering extracted 14,087 participants and 570,162 SNPs. Among these, we obtained the genotype data of the above-mentioned 431 general adults from the Kyoto J-MICC study. From the genotype data that passed quality control filtering, we identified rs4244285 (c.681G>A in exon 5), rs4986893 (c.636G>A in exon 4) [28,29], and rs1057910 (c.1075A>C in exon 7, CYP2C9*3), which decreases CYP2C9 activity [30]. Genotype imputation was performed using SHAPEIT2 [31] and Minimac3 [32] software based on the cosmopolitan reference panel of the 1000 Genomes Project (phase 3) [33]. We identified rs11188072 (c.−3402C>T in 5′-flanking region) and rs12248560 (c.−806C>T in 5′-flanking region) with imputation quality scores R^2^ ≥ 0.98. We confirmed that complete linkage existed between c.−3402T and c.−806T (r^2^ = 1) [6,7], and that c.−3402T, c.−806T, c.636A, and c.681A were mutually exclusive. Next, we defined the following four haplotypes: CYP2C19*1 (c.−3402C-c.−806C-c.636G-c.681G), CYP2C19*2 (c.−3402C-c.−806C-c.636G-c.681A), CYP2C19*3 (c.−3402C-c.−806C-c.636A-c.681G) and CYP2C19*17 (c.−3402T-c.−806T-c.636G-c.681G). Finally, participants were classified into extensive metabolizers (EM: CYP2C19*1/*1 or CYP2C19*1/*17), intermediate metabolizers (IM: CYP2C19*1/*2, CYP2C19*1/*3, CYP2C19*2/*17 or CYP2C19*3/*17), and poor metabolizers (PM: CYP2C19*2/*2, CYP2C19*2/*3 or CYP2C19*3/*3). Because CYP2C19 shares substantial substrate overlap with CYP2C9 due to the high structural homology within the CYP2C subfamily, we examined the specificity of candidate compounds as CYP2C19 biomarkers by performing genotyping for CYP2C9. When the CYP2C9*3 allele was not detected, the test allele was designated CYP2C9*1. Participants were further categorized as CYP2C9*3 allele carriers (CYP2C9*1/*3 or CYP2C9*3/*3) or non-carriers (CYP2C9*1/*1).

For the 255 patients, total DNA was extracted from 400 μL of whole blood sample using the Maxwell^®^ 16 DNA Purification Kit (Promega, Tokyo, Japan). Each sample was analyzed for the SNP rs4244285 (c.681G>A in exon 5), rs4986893 (c.636G>A in exon 4), rs11188072 (c.-3402C>T in 5′-flanking region), rs12248560 (c.-806C>T in 5′-flanking region), and rs1057910 (c.1075A>C in exon 7). Allelic discrimination reaction was carried out using a TaqMan^®^ genotyping assay (Thermo Fisher Scientific, Waltham, MA, USA) with a LightCycler Nano System (Roche Applied Science, Penzberg, Germany). The patients were classified into CYP2C19 EM, IM, and PM based on the presence or absence of the CYP2C19*2, CYP2C19*3, and CYP2C19*17 alleles. They were also classified into CYP2C9*3 allele carriers and non-carriers, as described above.

### 2.4. Plasma Sample Pretreatment

The plasma samples were pretreated by SPE using Oasis^®^ MAX μElution plate and Oasis^®^ PRiME MCX μElution plate, and by liquid-liquid extraction (LLE) to reduce matrix effects and allow global analysis of metabolites. In SPE using the Oasis^®^ MAX μElution plate, 50 μL of plasma sample was mixed with 100 μL of 5% ammonium hydroxide, vortexed, and centrifuged (5 min at 13,000 *g*, 10 °C). A µElution plate was pre-conditioned and equilibrated with 200 µL of methanol and water before use. One hundred and twenty μL of sample was loaded into a µElution plate, washed with 200 µL of 5% ammonium hydroxide and 200 µL of methanol, and eluted with 50 µL of methanol containing 2% formic acid into a 96-well collection plate. Each sample extract was diluted with 50 µL of water.

In SPE using an Oasis^®^ PRiME MCX μElution plate, 50 μL of plasma sample was mixed with 100 μL of 4% phosphoric acid, vortexed, and centrifuged (5 min at 13,000 *g*, 10 °C). One hundred and twenty μL of sample was loaded into a µElution plate, washed with 200 µL of 2% formic acid and 200 µL of methanol, and eluted with 50 µL of methanol containing 5% ammonium hydroxide into a 96-well collection plate. Each sample extract was diluted with 50 µL of water.

In LLE, 50 μL of plasma sample was mixed with 200 µL of chloroform/methanol (2/1, *v*/*v*) in a glass tube, vortexed, and centrifuged (5 min at 5000 *g*, 25 °C). One hundred μL of the lower organic phase was collected into a new glass tube and evaporated to dryness under an N_2_ gas stream at 40 °C. The residue was reconstituted with 250 μL of isopropanol/acetonitrile/water (2/1/1, *v*/*v*/*v*). All samples were vortex mixed and transferred to a 96-well collection plate.

Reference samples were prepared by mixing pretreated samples in a 96-well collection plate. The collection plate was sealed with an adhesive plate seal and set on the sample manager for direct injection into the ultra-performance liquid chromatography coupled to quadrupole time-of-flight mass spectrometry (UPLC-QTOF/MS) system.

### 2.5. Untargeted Metabolite Analysis

Untargeted metabolite analyses were performed using the UPLC-QTOF/MS system (Waters) consisting of a Waters Acquity Premier system and a Waters Xevo G2-XS Q-TOF mass spectrometer. A 5-μL aliquot of pretreated sample was injected into an ACQUITY UPLC^®^ BEH C18 column (1.7 μm, 2.1 mm × 100 mm, hydrophobic column, Waters) and an ACQUITY UPLC^®^ BEH HILIC column (1.7 μm, 2.1 mm × 100 mm, hydrophilic column, Waters) at 40 °C. Eluent A was 2 mM ammonium formate/acetonitrile (95/5, *v*/*v*) containing 0.1% formic acid. Eluent B was 2 mM ammonium formate/acetonitrile (5/95, *v*/*v*) containing 0.1% formic acid. The flow rate was 0.4 mL/min. The gradient program for the hydrophobic column was set as follows: 5% B (0 min to 0.5 min), 5% to 95% B (0.5 min to 12 min), 95% B (12 min to 16 min), 95% to 5% B (16 min to 16.01 min), and 5% B (16.01 min to 20 min). Gradient program for hydrophilic column was: 95% B (0 min to 0.5 min), 95% to 50% B (0.5 min to 10 min), 50% to 5% B (10 min to 10.01 min), 5% B (10.01 min to 14 min), 5% to 95% B (14 min to 14.01 min) and 95% B (14.01 min to 17.5 min). The parameters in positive ionization mode were: capillary voltage 3.0 kV, sampling cone voltage 30 V, cone gas flow 100 L/h, source temperature 150 °C, desolvation temperature 450 °C, and desolvation gas flow 800 L/h. The parameters in negative ionization mode were: capillary voltage 2.8 kV, sampling cone voltage 40 V, cone gas flow 50 L/h, source temperature 150 °C, desolvation temperature 500 °C, and desolvation gas flow 1000 L/h. Data were acquired using MS^E^ continuum mode in both positive and negative ion modes of electrospray ionization, and mass spectra were acquired for the mass-to-charge ratio (*m*/*z*) range of 100–1500 Da.

### 2.6. Data Treatment and Statistical Analysis of Untargeted Metabolome Analysis

The UPLC-QTOF/MS data were collected using MassLynx V4.2 (Waters). Untargeted data analyses were performed for 24 analytical units (2 participant cohorts × 3 sample pretreatments × 2 analytical columns × 2 ionization modes) using Progenesis^®^ QI 3.0 software (Waters). Metabolic profiles in CYP2C19 EM, IM, and PM were constructed by processing peak alignment, group creation, peak picking, normalization, and deconvolution. For peak alignment, the software selected the most appropriate measured data from among the reference samples. The reference data of each analytical unit were used to adjust retention time [34]. The following adduct ions were selected in the peak picking section: [M+H]^+^, [M+Na]^+^, and [M+NH_4_]^+^ in positive ionization mode, and [M−H]^−^ in negative ionization mode. For normalization, reference data were automatically selected by the software from among all measured data, and the reference data were used for intensity normalization of detected ions [35]. The deconvoluted ions were created and labeled as compounds having unique retention time and *m*/*z* [36]. These metabolic profiles were analyzed statistically.

Data from the general adult and patient cohorts were used to search for novel endogenous biomarkers for predicting CYP2C19 activity. To select endogenous biomarkers with good intensity, compounds having zero intensity in >20% of all participants were excluded [20,22]. Analysis of variance (ANOVA) was performed using Progenesis^®^ QI 3.0 software to identify compounds with normalized abundance significantly different between EM and PM. A *p*-value less than 0.05 was considered statistically significant. Compounds that showed significant differences between EM and PM by ANOVA were further subjected to orthogonal partial least squares-discriminant analysis (OPLS-DA) using EZinfo^®^ 3.0.3 software (Umetrics, Umeå, Sweden). OPLS-DA was used to prioritize variables contributing to EM−PM differentiation rather than to construct a predictive classification model. Data were Pareto-scaled without transformation before OPLS-DA. The performance of the OPLS-DA models was evaluated using seven-fold cross-validation. Model fit was assessed using the cumulative fraction of the variation in X and Y explained by the model [R^2^X(cum) and R^2^Y(cum), respectively], whereas predictive ability was evaluated using the cumulative Q^2^[Q^2^(cum)] obtained by cross-validation. Variable importance in the projection (VIP) value in OPLS–DA is an index that summarizes the importance of each variable in group separation, and a higher VIP value is interpreted as having greater contribution to group separation [37]. In this study, the compounds with VIP > 1.0 were interpreted as contributing to group separation of EM and PM and screened as candidate compounds predicting CYP2C19 activity.

### 2.7. Statistical Comparison of Candidate Compounds Predicting CYP2C19 Activity Between Phenotypes

Normalized abundance of candidate compounds was compared between CYP2C19 EM, IM, and PM and between CYP2C9*3 allele carriers and non-carriers. Zero values were defined as low signal intensity below the detection limit and were replaced by half of the minimum value detected [38]. Then, all data were transformed to natural logarithms. Data normality was analyzed by the Shapiro-Wilk test. Differences in log_-_normalized abundance of candidate compounds for predicting CYP2C19 activity between CYP2C19 EM, IM, and PM were compared by one-way ANOVA followed by Tukey’s post hoc test or Kruskal-Wallis test followed by Dunn’s post hoc test. To evaluate the specificity of candidate compounds as CYP2C19 biomarkers, differences in log_-_normalized abundance of candidate compounds between CYP2C9*3 allele carriers and non-carriers were compared by Welch’s *t*-test or the Mann-Whitney *U* test. Statistical analyses were performed using GraphPad Prism 10 (GraphPad Software, Boston, MA, USA). A *p*-value below 0.05 was considered statistically significant.

### 2.8. Identification of Candidate Compounds for Predicting CYP2C19 Activity

This study was designed as an exploratory metabolomics analysis, and compound confirmation using authentic reference standards was not performed. Metabolite identification was conducted according to the Metabolomics Standards Initiative (MSI) guidelines. In the absence of structural confirmation, the metabolite identification levels for the candidate metabolites were limited to MSI level 2 (putatively annotated compound), level 3 (putatively characterized compound class), or level 4 (unknown compound) [39]. Databases containing compound information from the Human Metabolome Database (HMDB) [40] and LipidBlast [41] were used to identify candidate compounds for predicting CYP2C19 activity. The precursor tolerance and theoretical fragment tolerance were set at 5 ppm for HMDB and LipidBlast. For each identified compound, “Score”, “Fragmentation score”, “Mass error,” and “Isotope similarity” were calculated by Progenesis^®^ QI 3.0 software. The “Score” is calculated based on the mean contributions of fragmentation score, mass error, and isotope similarity. This score is regarded as a parameter to evaluate the quality of compound identification [42]. In this study, the compound with the highest score was accepted when multiple compounds were identified.

### 2.9. Comparison of Predictive Performance of Candidate Compounds by Covariate-Adjusted ROC Analysis

To examine whether each candidate compound provides additional value for predicting CYP2C19 PM status beyond known determinants of CYP2C19 activity (age, liver function, renal function, and inflammatory status), ROC analysis was performed using a model adjusted for these covariates. To account for potential differences between the general adult and patient cohorts, cohort was additionally included as a covariate in the logistic regression models. A base logistic regression model (base model) was constructed using age, ALT, C-reactive protein (CRP), eGFR, and cohort as covariates to estimate the probability of being a PM. Candidate compound models were then constructed by incorporating the log_-_normalized abundance of each candidate compound into the base model. ROC curves were generated from the predicted probabilities, and apparent area under the curve (AUC) of candidate compound models was compared with that of the base model using the DeLong test. Internal validation of the logistic regression models was performed using bootstrap resampling with 1000 repetitions. For each bootstrap resample, the model was refitted, and its AUC was evaluated in both the bootstrap sample and the original dataset. Optimism was calculated as the difference between these AUCs, and the mean optimism across bootstrap resamples was subtracted from the apparent AUC to obtain the optimism-corrected AUC. Statistical analyses and graph generation were performed using R software version 4.5.2 (http://www.r-project.org), and adjusted *p*-values using the Holm method below 0.05 were considered statistically significant.

## 3. Results

### 3.1. Participant Characteristics

Table 1 shows the participant characteristics and laboratory data. Of the 686 participants (431 general adults and 255 patients), 233 were classified into CYP2C19 EM, 315 into IM, and 138 into PM. No significant differences in participant characteristics and laboratory data were observed among the three groups. The allelic frequencies were 0.55 for CYP2C19*1, 0.32 for CYP2C19*2, 0.11 for CYP2C19*3, and 0.017 for CYP2C19*17, and there was no participant homozygous for CYP2C19*17. Table S1 and Table S2 show the participant characteristics and laboratory data of the general adult cohort and the patient cohort, respectively. Table S3 shows the underlying diseases in the patient cohort. The primary underlying diseases were kidney disease, cancer, and pulmonary aspergillosis.

### 3.2. Search for Candidate Compounds as CYP2C19 Biomarkers

Table 2 shows the numbers of compounds detected by untargeted metabolite analysis for 12 analytical units. For each analytical unit, ANOVA was conducted on all the detected compounds to find those with significantly different normalized abundance between CYP2C19 EM and PM, and the detected compounds were further subjected to OPLS-DA. The OPLS-DA models showed low overall explanatory and predictive performance, with R^2^Y(cum) values ranging from 0.01 to 0.07 and Q^2^(cum) values ranging from 0.00 to 0.05, as shown in Table S4. Seventy-one compounds had VIP scores > 1.0 in OPLS-DA, and VIP plots are shown in Figure S1. Normalized abundance of the 71 compounds was compared between EM, IM, and PM, and nine compounds were significantly different among the three groups, as shown in Table S5. These nine compounds were considered candidate compounds for predicting CYP2C19 activity.

### 3.3. Identification of Candidate Compounds for Predicting CYP2C19 Activity

We attempted to identify the 9 candidate compounds described above by searching databases from HMDB and LipidBlast. One compound with *m*/*z* 384.0631 was excluded from subsequent analyses because it was putatively annotated as a metabolite of lansoprazole (MSI level 2). Table 3 summarizes the characteristics of the remaining 8 candidate compounds for predicting CYP2C19 activity. Database searches resulted in the assignment of putatively characterized compound classes (MSI level 3) for 3 candidate compounds: Compound A (*m*/*z* 811.5988) was classified as a glycerophospholipid or sphingolipid, Compound B (*m*/*z* 303.2319) as a fatty acyl or prenol lipid, and Compound C (*m*/*z* 902.5404) as a fatty acyl. Putative candidate compounds for Compounds A–C obtained by searching databases are listed in Table S6. However, the search failed to identify characteristics for compound classes for Compounds D–H (*m*/*z* 792.5744, 653.3021, 449.3238, 314.2309, and 160.1342, respectively), and these compounds were therefore assigned to MSI level 4.

### 3.4. Comparison of Normalized Abundance of Candidate Compounds as CYP2C19 Biomarker

**Figure 1:**
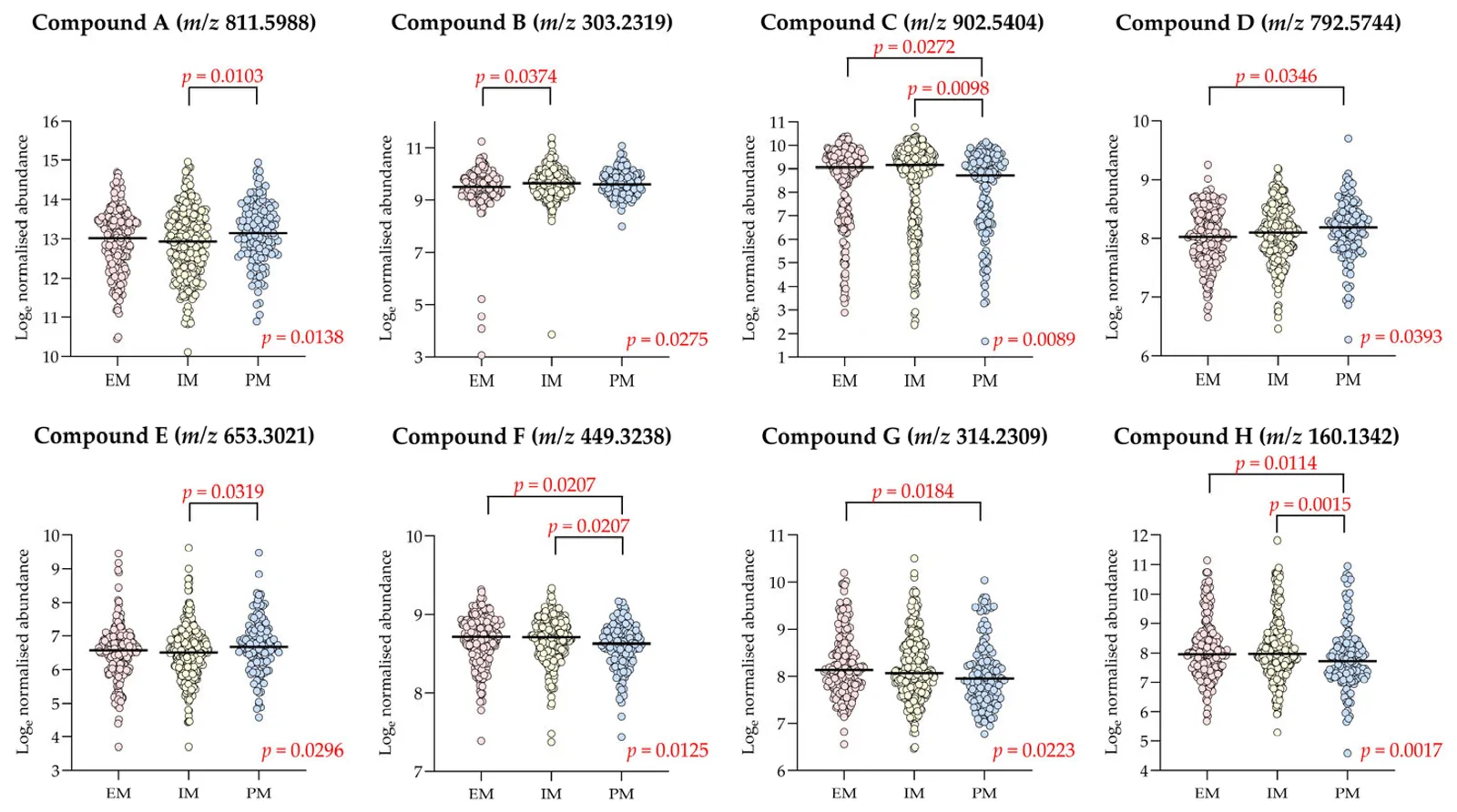
Comparison of log_-_normalized abundance of compounds A–H between CYP2C19 extensive metabolizers, intermediate metabolizers, and poor metabolizers. Data normality was analyzed by Shapiro-Wilk test. Statistical analyses were performed using one-way ANOVA followed by Tukey’s post hoc test, or the Kruskal-Wallis test followed by Dunn’s post hoc test. The horizontal line represents the mean or median value. A *p*-value less than 0.05 was considered statistically significant. EM, extensive metabolizers; IM, intermediate metabolizers; PM, poor metabolizers.

Figure 1 compares log_-_normalized abundance of Compounds A–H among CYP2C19 EM, IM, and PM. Significant differences in normalized abundance among the three groups were observed for all 8 compounds. The post hoc test showed significantly higher normalized abundance in EM or IM compared to PM for compounds with *m*/*z* 160.1342, 314.2309, 449.3238, and 902.5404 (a fatty acyl). On the other hand, normalized abundance was significantly lower in EM or IM compared to PM for compounds with *m*/*z* 653.3021, 792.5744, and 811.5988 (a glycerophospholipid or sphingolipid). For the compound with *m*/*z* 303.2319 (a fatty acyl or prenol lipid), normalized abundance was significantly lower in EM compared to IM.

Figure S2 compares log_-_normalized abundance of Compounds A–H between CYP2C9*3 allele carriers and non-carriers. Although the normalized abundance of Compound A was significantly higher in CYP2C9*3 allele carriers than in non-carriers, no significant differences were observed for Compounds B to H.

### 3.5. Comparison of Predicting Performance of Candidate Compounds by Covariate-Adjusted ROC Analysis

**Figure 2:**
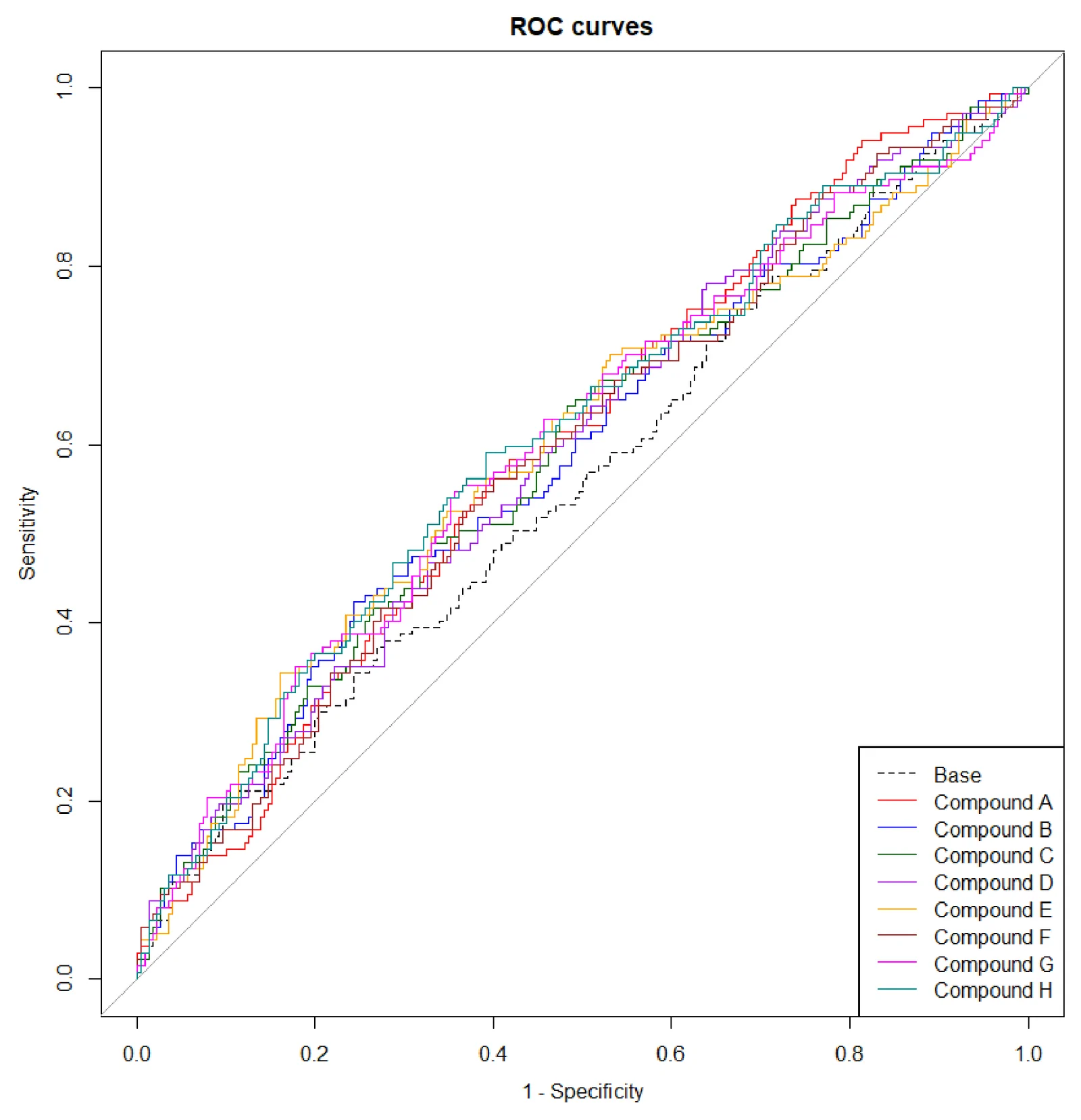
Receiver operating characteristic curves of the base model and the candidate compound models for the prediction of CYP2C19 PM status.

Figure 2 shows the ROC curves of the base model and the candidate compound models for predicting CYP2C19 PM status. Table 4 summarizes the apparent AUC with 95% confidence intervals, differences in apparent AUC from the base model (ΔAUC), mean optimism, and optimism-corrected AUC. Table S7 summarizes the optimal cutoff values, sensitivity, and specificity in each ROC analysis. Although the apparent AUCs of all candidate compound models were higher than that of the base model, no significant difference in apparent AUC was observed between the base model and each candidate compound model. Internal validation using 1000 bootstrap resamples indicated mean optimism of 0.035–0.042. The optimism-corrected AUC was 0.51 for the base model and ranged from 0.55 to 0.57 for the candidate compound models. Compound H with *m*/*z* 160.1342 showed the highest apparent AUC, ΔAUC, and optimism-corrected AUC.

## 4. Discussion

In this study, we searched for novel endogenous biomarkers in human plasma for predicting CYP2C19 activity, using CYP2C19 gene polymorphism data combined with untargeted metabolomic analysis results. The analyses yielded eight candidate compounds associated with CYP2C19 phenotype, comprising three putatively characterized compound classes (MSI level 3): glycerophospholipid/sphingolipid, fatty acyl, and fatty acyl/prenol lipid; and five unknown compounds (MSI level 4). The normalized abundance of all candidate compounds differed significantly among CYP2C19 EM, IM, and PM groups, suggesting that these compounds may reflect interindividual variability in CYP2C19 activity. In addition, covariate-adjusted ROC analysis demonstrated that all candidate compounds increased the apparent AUC and optimism-corrected AUC numerically beyond that of the base model, although none significantly improved the discrimination of CYP2C19 PM status. Among the candidate compounds, the compound with *m*/*z* 160.1342 showed the highest apparent AUC, ΔAUC, and optimism-corrected AUC. To the best of our knowledge, this is the first reported study to explore endogenous biomarkers of CYP2C19 activity in human plasma using untargeted metabolomics.

To minimize matrix effects and allow global analysis of metabolites, we pretreated human plasma samples using SPE with MAX (polymeric reversed-phase/strong anion exchange mixed-mode sorbent selective for acidic compounds) and MCX (polymeric reversed-phase/strong cation exchange mixed-mode sorbent selective for basic compounds), and also employed LLE for compounds not extracted by SPE. Using this approach, our search for CYP2C19 biomarkers yielded eight candidate compounds, including glycerophospholipid, sphingolipid, fatty acyl, and prenol lipid classes. The compounds with *m*/*z* 160.1342, 314.2309, 449.3238, and 902.5404 (a fatty acyl) may represent metabolites formed by CYP2C19, because their normalized abundance was significantly higher in EM or IM than in PM. In contrast, the compounds with *m*/*z* 653.3021, 792.5744, and 811.5988 (a glycerophospholipid or sphingolipid) may represent parent compounds metabolized by CYP2C19, because their normalized abundance was significantly higher in PM than in EM or IM. Lipid metabolism is regulated by multiple cytochrome P450 enzymes, including members of the CYP2C subfamily involved in the metabolism of endogenous fatty acids and lipid mediators [43]. Therefore, the association between the candidate lipid-related compounds and CYP2C19 phenotype may reflect direct or indirect effects of CYP2C19-mediated metabolic pathways. However, because all candidate compounds were assigned to MSI level 3 or 4 only, the biological mechanisms underlying their association with CYP2C19 phenotype remain unclear. Therefore, whether these compounds represent direct CYP2C19 substrates, CYP2C19-generated metabolites, or indirect downstream products associated with CYP2C19 activity remains to be investigated. An untargeted metabolomics approach is superior in detecting diverse metabolites, but elucidation of the chemical structures of all the detected metabolites remains a major challenge [44]. For example, using global metabolomics, Tay-Sontheimer et al. [45] detected a positive ion M1 with *m*/*z* 444.3102 as an endogenous urinary biomarker of CYP2D6 activity, and subsequently Behrle et al. [46] succeeded in isolating, purifying, and identifying M1 as 3,4-*seco*-solanidine-3,4-dioic acid from human urine, using several chromatographic techniques, mass spectrometry, and nuclear magnetic resonance. In our study, the available biological samples were limited to 150 µL of human plasma, which was inadequate for compound purification and characterization. Although structural identification of the candidate compounds is beyond the scope of this study, elucidating the structures of these compounds may validate the usefulness of these compounds as biomarkers for CYP2C19 activity.

The performance of candidate compounds for predicting CYP2C19 PM status was evaluated by ROC analysis adjusted for covariates of age, ALT, eGFR, CRP, and cohort, which are potential determinants of compound abundance and CYP2C19 activity. Although none of the candidate compound models showed significantly higher apparent AUC compared to the base model, all candidate compounds consistently increased the apparent AUC and optimism-corrected AUC of the base model. Although the compound with *m*/*z* 160.1342 showed the highest predictive performance, its apparent AUC of 0.61 and optimism-corrected AUC of 0.57 suggest limited discriminative ability [47]. These findings suggest that the identified compounds alone are insufficient as standalone biomarkers for predicting CYP2C19 phenotype. Nevertheless, the consistent increases in apparent AUC and optimism-corrected AUC across all candidate compounds indicate that these metabolites may contain information related to CYP2C19 activity and could contribute to future biomarker panels. Several factors may explain the modest predictive performance observed in the present study. First, although the study population included both general adults and patients with various underlying diseases, including kidney disease, cancer, and pulmonary aspergillosis, these underlying diseases were not included as covariates in the ROC analysis. Disease-related pathological alterations may affect endogenous metabolite concentrations independent of CYP2C19 activity, thereby increasing overlap in compound abundance between CYP2C19 phenotypes and reducing discriminatory performance in ROC analysis. Second, despite adjustment for age, ALT, eGFR, CRP, and cohort, residual confounding due to unmeasured physiological factors and environmental factors, including co-administered drugs, cannot be excluded. Third, endogenous metabolites are often regulated by multiple metabolic pathways, transporters, and excretory processes, reducing their specificity for a single CYP enzyme. For example, the normalized abundance of the compound with *m*/*z* 811.5988 was significantly higher in CYP2C9*3 allele carriers than in non-carriers, suggesting that at least some compounds may not be specific biomarkers of CYP2C19 activity.

If the compounds with *m*/*z* 160.1342, 314.2309, 449.3238, and 902.5404 (a fatty acyl) are CYP2C19-generated metabolites, their parent compounds would be expected to have higher abundance in PM than in EM. Likewise, if the compounds with *m*/*z* 653.3021, 792.5744, and 811.5988 (a glycerophospholipid or sphingolipid) are CYP2C19 substrates, their metabolites would be expected to be more abundant in EM than in PM. However, we did not identify candidate parents or metabolites that differed significantly among genotype-based phenotypes. For quantification of CYP activity using endogenous biomarkers, metabolites produced by specific CYPs or metabolite-to-parent ratios are commonly used [17]. Solanidine is a potato alkaloid proposed as a CYP2D6 biomarker by a global metabolomics approach. Several studies have demonstrated the associations of metabolite-to-solanidine ratios with CYP2D6 phenotypes [21,48,49], as well as with metabolism of CYP2D6 substrates, such as risperidone [50], metoprolol [49], and tamoxifen [51]. Hence, evaluating metabolite-to-parent ratios for the candidate CYP2C19 biomarkers identified in this study may improve the sensitivity of CYP2C19 activity quantification compared with measuring candidate compounds alone.

This study has some limitations. First, the candidate compounds were identified based on database searches and were not confirmed using authentic reference standards. Therefore, all compounds could only be assigned to MSI level 3 or 4. In addition, although OPLS-DA was used only as an exploratory compound-selection step rather than as a predictive classifier of CYP2C19 phenotype, the low R^2^Y and Q^2^ values indicated limited model performance. Therefore, the VIP-based prioritization of candidate compounds should be interpreted cautiously. Furthermore, although bootstrap internal validation was performed to quantify and correct for optimism in the logistic regression models, the candidate compounds had been selected using the same dataset before model evaluation. Thus, the bootstrap procedure did not account for potential optimism arising from the preceding compound-selection process. Independent validation of these candidate metabolites in an external cohort is therefore warranted. Second, different genotyping methods were used in the general adult and patient cohorts. However, both methods targeted the same functional CYP2C19 and CYP2C9 variants, and the imputed variants used for genotype assignment showed excellent imputation quality (R^2^ ≥ 0.98), suggesting minimal impact on genotype classification. Third, although general adult participants were selected based on BMI, ALT, total bilirubin, and eGFR, individuals with diseases such as hypertension, diabetes mellitus, and dyslipidemia may have been included. Furthermore, although patient participants were selected based on similar criteria, the patient cohort encompassed a wide range of diseases. Finally, the cross-sectional design precluded direct assessment of the relationship between candidate compound abundance and measured CYP2C19 activity determined using probe substrates such as omeprazole.

## 5. Conclusions

Untargeted metabolomics combined with CYP2C19 gene polymorphism analyses yielded eight candidate compounds associated with CYP2C19 phenotype. Despite their modest performance for predicting CYP2C19 PM status, these compounds may provide novel insights into endogenous biomarkers of CYP2C19 activity. Further structural characterization, quantitative validation, and external validation are warranted.

## Figures and Tables

**Table 1:** Demographic and laboratory data analyzed in the study.

	CYP2C19 Extensive Metabolizer	CYP2C19 Intermediate Metabolizer	CYP2C19 Poor Metabolizer
Number of participants; n	233	315	138
Sex (male/female); n (%)	110 (47.2)/123 (52.8)	141 (44.8)/174 (55.2)	68 (49.3)/70 (50.7)
Age (years)	58.0 (49.0−68.0)	59.0 (48.0−67.5)	59.5 (48.0−67.0)
Body mass index (kg/m^2^)	21.5 (19.1−23.6)	21.4 (19.3−23.4)	21.7 (19.9−23.9)
Estimated glomerular filtration rate (mL/min/1.73 m^2^)	70.9 (63.0−81.9)	71.6 (62.6−82.1)	73.0 (63.9−83.1)
Alanine aminotransferase (IU/L)	16.0 (11.3−21.0)	15.0 (11.7−22.0)	16.0 (12.0−24.7)
Total bilirubin (mg/dL)	0.70 (0.50−0.90)	0.70 (0.58−0.87)	0.70 (0.54−0.90)
C-reactive protein (mg/dL)	0.040 (0.015−0.17)	0.039 (0.018−0.17)	0.039 (0.013−0.14)
CYP2C19 genotype; n (%)			
CYP2C19*1/*1	133 (90.5)	–	
CYP2C19*1/*17	14 (9.5)	–	
CYP2C19*1/*2	–	138 (70.4)	–
CYP2C19*1/*3	–	55 (28.1)	–
CYP2C19*2/*17	–	2 (1.0)	–
CYP2C19*3/*17	–	1 (0.5)	–
CYP2C19*2/*2	–	–	50 (56.8)
CYP2C19*2/*3	–	–	34 (38.6)
CYP2C19*3/*3	–	–	4 (4.5)

Data are expressed as numbers (%) for categorical variables and median (interquartile range) for continuous variables. Statistical comparisons were conducted between CYP2C19 extensive metabolizers, intermediate metabolizers, and poor metabolizers. Sex ratio was compared by the chi-square test. Normality of the data was tested by the Shapiro-Wilk test, and analyses for continuous variables were performed by the Kruskal-Wallis test. A *p*-value less than 0.05 was considered statistically significant. C-reactive protein values were from participants whose data were available in electronic medical records (extensive metabolizer, n = 232; intermediate metabolizer, n = 311; poor metabolizer, n = 138).

**Table 2:** Compounds detected by untargeted metabolite analysis for 12 analytical units.

Pretreatment	Column	Ionization Mode	No. of Compounds Detected	No. of Compounds After Excluding Compounds with Zero Intensity in >20% of All Participants	No. of Compounds with Significant Differences in Normalized Abundance by ANOVA *	No. of Compounds with VIP > 1.0 in OPLS-DA
LLE	hydrophobic	positive	6326	1770	6	1
		negative	7092	1802	23	3
	hydrophilic	positive	7231	1770	15	3
		negative	6680	2318	25	5
SPE (MAX)	hydrophobic	positive	11,628	2561	24	2
		negative	9851	2409	42	9
	hydrophilic	positive	7178	2203	20	5
		negative	5756	1946	2	1
SPE (MCX)	hydrophobic	positive	14,609	3652	57	12
		negative	11,686	2728	47	12
	hydrophilic	positive	7349	2098	65	11
		negative	6234	2032	35	7

Data are expressed as numbers. * Analysis of variance was conducted between CYP2C19 extensive metabolizers and poor metabolizers. A *p*-value less than 0.05 was considered statistically significant. ANOVA, analysis of variance; LLE, liquid-liquid extraction; MAX, reversed-phase/strong anion exchange mixed-mode; MCX, reversed-phase/strong cation exchange mixed-mode; OPLS-DA, orthogonal partial least squares-discriminant analysis; SPE, solid-phase extraction; VIP, variable importance in the projection.

**Table 3:** Characteristics of the candidate compounds for predicting CYP2C19 activity.

Compound Name	Pretreatment	Column	Ionization Mode	Retention Time (min)	*m*/*z*	Chemical Class	MSI Level
A	LLE	hydrophobic	positive	11.96	811.5988	glycerophospholipid, sphingolipid	3
B	LLE	hydrophobic	negative	11.37	303.2319	fatty acyl, prenol lipid	3
C	LLE	hydrophilic	negative	1.39	902.5404	fatty acyl	3
D	LLE	hydrophilic	negative	3.75	792.5744	–	4
E	SPE (MCX)	hydrophobic	negative	9.77	653.3021	–	4
F	SPE (MCX)	hydrophobic	negative	11.70	449.3238	–	4
G	SPE (MCX)	hydrophilic	positive	2.68	314.2309	–	4
H	SPE (MCX)	hydrophilic	positive	3.55	160.1342	–	4

LLE, liquid-liquid extraction; MCX, reversed-phase/strong cation exchange mixed-mode; MSI, the Metabolomics Standards Initiative; *m*/*z*, mass-to-charge ratio; SPE, solid-phase extraction.

**Table 4:** Validity of the candidate compounds for predicting CYP2C19 PM status.

Model	Apparent AUC (95% CI)	ΔAUC	*p*-Value	Holm-Adjusted *p*-Value	Mean Optimism	Optimism-Corrected AUC
Base	0.56 (0.49−0.62)	–	–	–	0.042	0.51
Compound A (*m*/*z* 811.5988)	0.60 (0.54−0.66)	0.044	0.1454	0.8723	0.035	0.56
Compound B (*m*/*z* 303.2319)	0.59 (0.53−0.65)	0.032	0.2160	0.8723	0.039	0.55
Compound C (*m*/*z* 902.5404)	0.59 (0.53−0.65)	0.036	0.1735	0.8723	0.038	0.55
Compound D (*m*/*z* 792.5744)	0.59 (0.53−0.65)	0.038	0.1608	0.8723	0.038	0.56
Compound E (*m*/*z* 653.3021)	0.60 (0.54−0.66)	0.042	0.1667	0.8723	0.039	0.56
Compound F (*m*/*z* 449.3238)	0.59 (0.53−0.65)	0.035	0.1923	0.8723	0.038	0.55
Compound G (*m*/*z* 314.2309)	0.60 (0.54−0.66)	0.047	0.1002	0.7013	0.038	0.56
Compound H (*m*/*z* 160.1342)	0.61 (0.55−0.67)	0.053	0.0779	0.6234	0.036	0.57

Only participants with C-reactive protein levels available were included in the receiver operating characteristic analysis (extensive metabolizer, n = 230; poor metabolizer, n = 137). The base model included age, alanine aminotransferase, C-reactive protein, estimated glomerular filtration rate, and cohort as covariates to estimate the probability of being a PM. Each candidate compound model was constructed by adding the corresponding log_-_normalized abundance of the candidate compound to the base model. Internal validation was performed using 1000 bootstrap resamples. Optimism-corrected AUC was calculated by subtracting the mean optimism from the apparent AUC. AUC, area under the curve; CI, confidence interval; ΔAUC, difference in apparent AUC from the base model. *p*-value: apparent AUC of each compound versus apparent AUC of the base model.

## Data Availability

The data that support the findings of this study are available on request from the corresponding author. The data are not publicly available due to privacy or ethical restrictions.

## Supplementary Materials

The following supporting information can be downloaded at: https://www.mdpi.com/article/10.3390/metabo16090670/s1. Table S1: Demographic and laboratory data of the general adult cohort analyzed in the study; Table S2: Demographic and laboratory data of the patient cohort analyzed in the study; Table S3: Underlying diseases of the patient cohort analyzed in the study; Table S4: Performance parameters of the OPLS-DA models; Figure S1: VIP plot of OPLS-DA for each analytical unit.; Table S5: Candidate compounds for predicting CYP2C19 activity.; Table S6: Putative candidate compounds as CYP2C19 biomarkers by searching databases; Figure S2: Comparison of log-normalized abundance of Compounds A–H between *CYP2C9*3* allele carriers and non-carriers; Table S7: Optimal cutoff values, sensitivity, and specificity in each receiver operating characteristic analysis.

## Abbreviations

The following abbreviations are used in this manuscript:

ALT	Alanine aminotransaminase
ANOVA	Analysis of variance
AUC	Area under the curve
BMI	Body mass index
CRP	C-reactive protein
CYP	Cytochrome P450
eGFR	Estimated glomerular filtration rate
EM	Extensive metabolizers
HMDB	the Human Metabolome Database
IM	Intermediate metabolizers
J-MICC Study	the Japan Multi-Institutional Collaborative Cohort Study
LLE	Liquid-liquid extraction
*m*/*z*	Mass-to-change ratio
MSI	the Metabolomics Standards Initiative
OPLS-DA	Orthogonal partial least squares–discriminant analysis
PM	Poor metabolizers
ROC	Receiver operating characteristic
SNPs	Single nucleotide polymorphisms
SPE	Solid-phase extraction
UPLC-QTOF/MS	Ultra-performance liquid chromatography coupled to quadrupole time-of-flight mass spectrometry
VIP	Variable importance in the projection
