# Supplementary material for: Search for Novel Biomarkers to Predict Cytochrome P450 2C19 Activity Using Untargeted Metabolomics of Human Plasma
Source: Metabolites. 2026 Sep 11;16(9):670. doi: 10.3390/metabo16090670 (PMC13609000; doi:10.3390/metabo16090670)
Supplement: Supplementary file 1 [file metabolites-16-00670-s001.zip › metabolites-4490978-supplementary.pdf]

Table S1. Demographic and laboratory data of the general adult cohort analyzed in the study.

	CYP2C19 extensive metabolizer	CYP2C19 intermediate metabolizer	CYP2C19 poor metabolizer
Number of participants; n	147	196	88
Sex (male/female); n (%)	50 (34.0)/97 (66.0)	68 (34.7)/128 (65.3)	33 (37.5)/55 (62.5)
Age (years)	53.0 (47.0–62.0)	56.5 (47.0–66.0)	54.0 (45.8–64.0)
Body mass index (kg/m^2)	21.7 (19.5–23.6)	21.8 (19.7–23.6)	21.7 (20.3–24.0)
Estimated glomerular filtration rate (mL/min/1.73 m^2)	74.1 (67.5–82.6)	73.7 (68.3–84.0)	75.5 (68.4–82.9)
Alanine aminotransferase (IU/L)	16.0 (12.0–20.0)	15.0 (12.0–20.3)	16.0 (13.8–23.0)
Total bilirubin (mg/dL)	0.70 (0.60–0.90)	0.70 (0.60–0.90)	0.70 (0.60–0.90)
CYP2C19 genotype; n (%)			
*CYP2C19*1/*1*	133 (90.5)	–	
*CYP2C19*1/*17*	14 (9.5)	–	
*CYP2C19*1/*2*	–	138 (70.4)	–
*CYP2C19*1/*3*	–	55 (28.1)	–
*CYP2C19*2/*17*	–	2 (1.0)	–
*CYP2C19*3/*17*	–	1 (0.5)	–
*CYP2C19*2/*2*	–	–	50 (56.8)
*CYP2C19*2/*3*	–	–	34 (38.6)
*CYP2C19*3/*3*	–	–	4 (4.5)

Data are expressed as number (%) for categorical variables and median (interquartile range) for continuous variables.

Table S2. Demographic and laboratory data of the patient cohort analyzed in the study.

	CYP2C19 extensive metabolizer	CYP2C19 intermediate metabolizer	CYP2C19 poor metabolizer
Number of participants; n	86	119	50
Sex (male/female); n (%)	60 (70.0)/26 (30.0)	73 (61.3)/46 (38.7)	35 (70.0)/15 (30.0)
Age (years)	67.0 (55.0–72.0)	64.0 (50.0–72.0)	62.0 (53.5–73.0)
Body mass index (kg/m^2)	21.3 (18.4–23.5)	20.5 (18.6–22.6)	21.2 (19.5–23.7)
Estimated glomerular filtration rate (mL/min/1.73 m^2)	63.4 (45.5–78.4)	58.5 (40.6–75.7)	63.1 (36.0–82.7)
Alanine aminotransferase (IU/L)	14.5 (10.8–23.3)	15.1 (10.0–24.0)	17.3 (11.1–25.9)
Total bilirubin (mg/dL)	0.60 (0.43–0.84)	0.62 (0.46–0.78)	0.60 (0.44–0.82)
CYP2C19 genotype; n (%)			
*CYP2C19*1/*1*	82 (95.3)	–	
*CYP2C19*1/*17*	4 (4.7)	–	
*CYP2C19*1/*2*	–	82 (68.9)	–
*CYP2C19*1/*3*	–	34 (28.6)	–
*CYP2C19*2/*17*	–	2 (1.7)	–
*CYP2C19*3/*17*	–	1 (0.8)	–
*CYP2C19*2/*2*	–	–	32 (64.0)
*CYP2C19*2/*3*	–	–	16 (32.0)
*CYP2C19*3/*3*	–	–	2 (4.0)

Data are expressed as number (%) for categorical variables and median (interquartile range) for continuous variables.

Table S3. Underlying diseases of the patient cohort analyzed in the study.

Underlying disease	Number of patients
Acute respiratory distress syndrome	1
Anal canal cancer	1
Arthritis	1
Ascending colon cancer	4
Atrial fibrillation	1
Atrial tachycardia	1
Carcinoma of unknown primary	3
Cholangiocarcinoma	3
Chronic glomerulonephritis	13
Chronic kidney disease	25
Chronic obstructive pulmonary disease	2
Chronic progressive pulmonary aspergillosis	11
Chronic pulmonary aspergillosis	7
Colon cancer	1
Congenital heart disease	1
Deep venous thrombosis	2
Dementia with Lewy body	1
Diabetic kidney disease	23
Double cancer	2
Esophageal cancer	11
Extensive disease small cell lung cancer	3
Gastric cancer	14
Gastric gastrointestinal stromal tumor	1
Gouty nephropathy	2
Idiopathic interstitial pneumonia	1
Idiopathic pulmonary fibrosis	1
Immunoglobulin a nephropathy	13
Immunoglobulin a nephropathy + Nephrosclerosis	1
Infected cyst	1
Infectious spondylitis	1
Interstitial nephritis	3
Intrahepatic cholangiocarcinoma	1
Limited disease small cell lung cancer	1
Liposarcoma	1
Lung adenocarcinoma	4
Lupus nephritis	1
Malignant melanoma of the esophagus	1
Malignant mesothelioma	3
Mediastinal tumor	2
Mixed neuroendocrine-non-neuroendocrine neoplasm of the colon	1
Nephrosclerosis	12
Nephrotic syndrome	4
Neuroendocrine carcinoma	3
Non-small cell lung cancer	4

Pancreatic body cancer	1
Pancreatic cancer	8
Pancreatic head carcinoma	5
Pancreatic neuroendocrine tumor	2
Pancreatic tail cancer	2
Parkinson's disease	4
Polycystic kidney	4
Pulmonary aspergilloma	3
Pulmonary aspergillosis	5
Pyelonephritis	3
Rectal cancer	7
Reflux nephropathy	1
Sigmoid colon cancer	2
Small bowel gastrointestinal stromal tumor	1
Small cell lung cancer	5
Soft tissue sarcoma	1
Squamous cell carcinoma of the lung	4
Takayasu arteritis	1
Thymoma	2
Traffic injury	1
Total	255

Table S4. Performance parameters of the OPLS-DA models.

Pretreatment	Column	Ionization mode	Predictive components	Orthogonal components	R^2X(cum)	R^2Y(cum)	Q^2(cum)
LLE	hydrophobic	positive	1	1	0.99	0.01	0.00
		negative	1	1	0.64	0.04	0.02
	hydrophilic	positive	1	1	0.73	0.05	0.03
		negative	1	1	0.48	0.07	0.05
SPE (MAX)	hydrophobic	positive	1	1	0.95	0.03	0.01
		negative	1	1	0.63	0.03	0.00
	hydrophilic	positive	1	1	0.73	0.03	0.02
		negative	1	1	1.00	0.01	0.00
SPE (MCX)	hydrophobic	positive	1	1	0.39	0.06	0.00
		negative	1	1	0.46	0.04	0.02
	hydrophilic	positive	1	1	0.69	0.02	0.01
		negative	1	1	0.69	0.02	0.01

Data are expressed as numbers or proportions. LLE, liquid-liquid extraction; MAX, reversed-phase/strong anion exchange mixed-mode; MCX, reversed-phase/strong cation exchange mixed-mode; OPLS-DA, orthogonal partial least squares-discriminant analysis; SPE, solid-phase extraction.

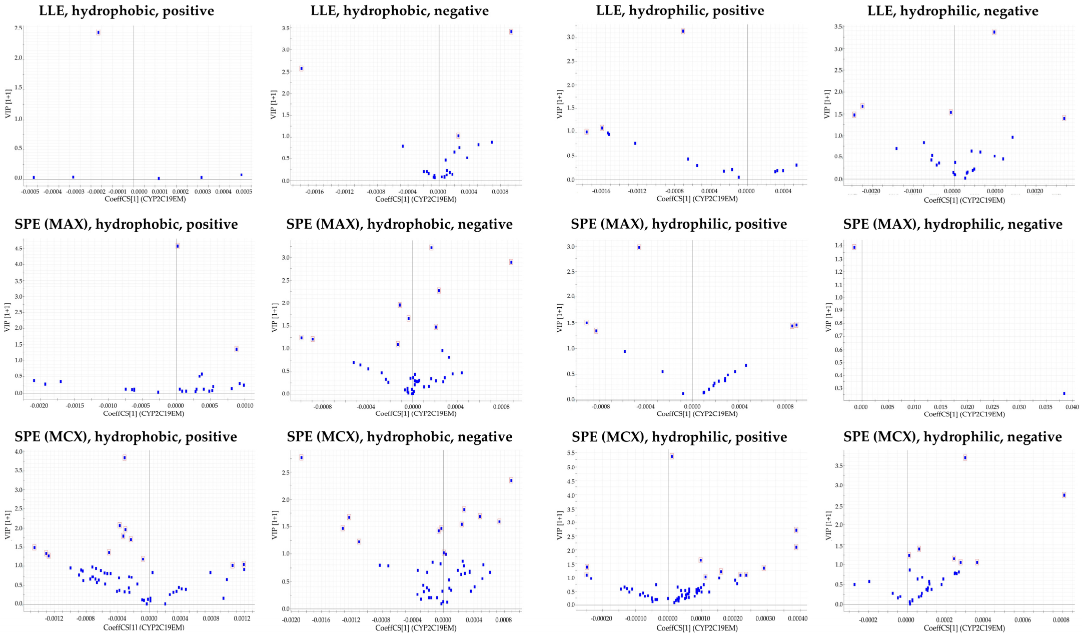

Figure S1. VIP plot of OPLS-DA for each analytical unit.

The y-axis is VIP score and the x-axis is partial least squares-coefficient. The red open square represents the compounds with VIP > 1.0. LLE, liquid-liquid extraction; MAX, reversed-phase/strong anion exchange mixed-mode; MCX, reversed-phase/strong cation exchange mixed-mode; OPLS-DA, orthogonal partial least squares-discriminant analysis; SPE, solid-phase extraction; VIP, variable importance in the projection.

Table S5. Candidate compounds for predicting CYP2C19 activity.

Pretreatment	Column	Ionization mode	Retention time (min)	*m/z*	*p*-value of ANOVA	VIP score of OPLS-DA	*p*-value of multiple comparison
LLE	hydrophobic	positive	<u>11.96</u>	<u>811.5988</u>	<u>0.0308</u>	<u>2.45</u>	<u>0.0138</u>
		negative	9.87	552.3672	0.0262	1.04	0.1100
			<u>11.37</u>	<u>303.2319</u>	<u>0.0216</u>	<u>2.56</u>	<u>0.0275</u>
			12.72	871.6748	0.0362	3.40	0.0554
	hydrophilic	positive	0.91	362.1201	0.0225	3.13	0.1223
			1.39	726.6257	0.0251	1.01	0.1395
			4.23	1476.2283	0.0174	1.09	0.0953
		negative	1.39	874.4975	0.0273	1.53	0.1415
			<u>1.39</u>	<u>902.5404</u>	<u>0.0187</u>	<u>3.36</u>	<u>0.0089</u>
			3.55	946.6873	0.0460	1.40	0.2062
			<u>3.75</u>	<u>792.5744</u>	<u>0.0174</u>	<u>1.47</u>	<u>0.0393</u>
			3.98	627.4092	0.0497	1.67	0.4605
SPE (MAX)	hydrophobic	positive	0.75	160.0618	0.0139	1.36	0.1304
			0.80	293.0989	0.0277	4.56	0.1050
		negative	0.70	313.0643	0.0447	1.10	0.2259
			0.70	605.1560	0.0235	2.90	0.2261
			0.70	621.1253	0.0270	2.27	0.3257
			0.70	913.2162	0.0422	1.48	0.3469
			0.75	247.0932	0.0424	1.66	0.0728
			0.75	273.0725	0.0301	1.96	0.1567
			0.75	291.2532	0.0431	3.22	0.0534
			0.75	335.0482	0.0257	1.24	0.1217
			1.29	195.0517	0.0398	1.21	0.1003
	hydrophilic	positive	0.75	638.5920	0.0375	1.34	0.4482
			0.75	844.7346	0.0369	1.50	0.4276
			1.09	331.0933	0.0368	2.97	0.2603
			5.20	132.0669	0.0444	1.45	0.2245
			5.20	247.0939	0.0325	1.44	0.1869
		negative	1.04	345.0735	0.0228	1.39	0.1145
SPE (MCX)	hydrophobic	positive	0.65	258.1115	0.0362	3.84	0.1096
			3.37	1142.0227	0.0211	1.49	0.0598
			3.37	1142.5242	0.0150	1.33	0.0640
			3.37	1288.0727	0.0407	1.27	0.1884
			5.30	749.6112	0.0277	1.18	0.1371
			5.30	936.0119	0.0375	1.70	0.1644
			5.30	936.2631	0.0264	1.95	0.1979
			5.30	936.5131	0.0085	2.06	0.2330
			5.30	936.7644	0.0245	1.80	0.2198
			5.48	1306.4876	0.0157	1.04	0.0575
			5.86	1215.0782	0.0437	1.37	0.1197
			10.36	546.3958	0.0188	1.02	0.0702
		negative	3.45	1250.5390	0.0319	1.47	0.1558
			4.67	1026.8621	0.0437	1.42	0.2455
			4.67	1027.1958	0.0481	1.46	0.2497
			<u>5.78</u>	<u>384.0631</u>	<u>0.0288</u>	<u>2.76</u>	<u>0.0386</u>
			<u>9.77</u>	<u>653.3021</u>	<u>0.0192</u>	<u>1.22</u>	<u>0.0296</u>
			11.32	429.2870	0.0466	1.54	0.1206
			11.70	443.3026	0.0244	1.81	0.0694
			<u>11.70</u>	<u>449.3238</u>	<u>0.0167</u>	<u>2.35</u>	<u>0.0125</u>
			11.96	303.2333	0.0054	1.67	0.0899

		12.01	349.2742	0.0410	1.02	0.1591
		12.34	299.2586	0.0139	1.59	0.1077
		13.63	233.1554	0.0300	1.69	0.1178
hydrophilic	positive	0.81	376.2604	0.0421	5.36	0.2178
		0.81	421.3185	0.0425	1.20	0.1837
		1.04	270.1495	0.0290	1.62	0.1499
		2.63	330.2629	0.0336	1.33	0.0885
		<u>2.68</u>	<u>314.2309</u>	<u>0.0163</u>	<u>1.02</u>	<u>0.0223</u>
		2.68	350.2081	0.0331	1.09	0.1845
		<u>3.55</u>	<u>160.1342</u>	<u>0.0064</u>	<u>1.09</u>	<u>0.0017</u>
		5.05	175.1201	0.0277	2.70	0.3907
		7.66	1377.2209	0.0409	1.37	0.2052
		12.54	128.9524	0.0345	1.09	0.1061
		12.62	141.9600	0.0283	2.08	0.0623
	negative	0.60	199.8521	0.0310	1.39	0.0740
		0.91	137.0247	0.0180	1.05	0.1715
		1.14	136.8924	0.0417	3.68	0.0718
		3.12	216.8544	0.0191	1.15	0.1722
		3.12	296.8210	0.0280	1.05	0.1918
		3.22	100.9316	0.0281	1.24	0.0617
		5.38	173.1045	0.0451	2.75	0.0822

Analysis of variance was conducted between CYP2C19 extensive metabolizer and poor metabolizer. Multiple comparison was conducted between CYP2C19 extensive metabolizer, intermediate metabolizer, and poor metabolizer. Compounds showing significant differences among the three groups are underlined. Normality of the data were tested by Shapiro-Wilk test. Parametric data were compared by 1-way analysis of variance followed by Tukey's post hoc test. Nonparametric data were compared by Kruskal-Wallis test followed by Dunn's post hoc test. A *p*-value less than 0.05 was considered statistically significant. ANOVA, analysis of variance; LLE, liquid-liquid extraction; MAX, reversed-phase/strong anion exchange mixed-mode; MCX, reversed-phase/strong cation exchange mixed-mode; *m*/*z*, mass-to-change ratio; OPLS-DA, orthogonal partial least squares-discriminant analysis; SPE, solid-phase extraction; VIP, variable importance in the projection.

Table S6. Putative candidate compounds as CYP2C19 biomarkers by searching databases.

Compound name	Description	Adducts	Formula	Score	Fragmentation score	Mass error (ppm)	Isotope similarity
A	SM(d20:1/20:4(8Z,11Z,14Z,17Z)-2OH(5S,6R))	M+H	$C_{45}H_{83}N_2O_8P$	30.7	0	3.5	57.7
	SM(d20:1/20:4(6Z,8E,10E,14Z)-2OH(5S,12R))	M+H	$C_{45}H_{83}N_2O_8P$	30.7	0	3.5	57.7
	SM(d20:1/20:4(6E,8Z,11Z,13E)-2OH(5S,15S))	M+H	$C_{45}H_{83}N_2O_8P$	30.7	0	3.5	57.7
	PE(20:4(5Z,8Z,11Z,14Z)/20:1(11Z))	$M+NH_4$	$C_{45}H_{80}NO_8P$	30.7	0.080	3.5	57.6
	PE(20:4(8Z,11Z,14Z,17Z)/20:1(11Z))	$M+NH_4$	$C_{45}H_{80}NO_8P$	30.7	0.080	3.5	57.6
	PE(22:4(7Z,10Z,13Z,16Z)/18:1(11Z))	$M+NH_4$	$C_{45}H_{80}NO_8P$	30.7	0.080	3.5	57.6
	PE(22:4(7Z,10Z,13Z,16Z)/18:1(9Z))	$M+NH_4$	$C_{45}H_{80}NO_8P$	30.7	0.080	3.5	57.6
	PE(20:1(11Z)/20:4(5Z,8Z,11Z,14Z))	$M+NH_4$	$C_{45}H_{80}NO_8P$	30.7	0.080	3.5	57.6
	PE(20:1(11Z)/20:4(8Z,11Z,14Z,17Z))	$M+NH_4$	$C_{45}H_{80}NO_8P$	30.7	0.080	3.5	57.6
	PE(18:1(9Z)/22:4(7Z,10Z,13Z,16Z))	$M+NH_4$	$C_{45}H_{80}NO_8P$	30.7	0.080	3.5	57.6
	PE(18:1(11Z)/22:4(7Z,10Z,13Z,16Z))	$M+NH_4$	$C_{45}H_{80}NO_8P$	30.7	0.080	3.5	57.6
	PE-NMe2(20:4(5Z,8Z,11Z,14Z)/18:1(11Z))	$M+NH_4$	$C_{45}H_{80}NO_8P$	30.7	0.080	3.5	57.6
	PE-NMe2(20:4(5Z,8Z,11Z,14Z)/18:1(9Z))	$M+NH_4$	$C_{45}H_{80}NO_8P$	30.7	0.080	3.5	57.6
	PE-NMe2(20:4(8Z,11Z,14Z,17Z)/18:1(11Z))	$M+NH_4$	$C_{45}H_{80}NO_8P$	30.7	0.080	3.5	57.6
	PE-NMe2(20:4(8Z,11Z,14Z,17Z)/18:1(9Z))	$M+NH_4$	$C_{45}H_{80}NO_8P$	30.7	0.080	3.5	57.6
	PE-NMe2(22:4(7Z,10Z,13Z,16Z)/16:1(9Z))	$M+NH_4$	$C_{45}H_{80}NO_8P$	30.7	0.080	3.5	57.6
	PE-NMe2(18:1(11Z)/20:4(8Z,11Z,14Z,17Z))	$M+NH_4$	$C_{45}H_{80}NO_8P$	30.7	0.080	3.5	57.6
	PE-NMe2(18:1(9Z)/20:4(8Z,11Z,14Z,17Z))	$M+NH_4$	$C_{45}H_{80}NO_8P$	30.7	0.080	3.5	57.6
	PE-NMe2(16:1(9Z)/22:4(7Z,10Z,13Z,16Z))	$M+NH_4$	$C_{45}H_{80}NO_8P$	30.7	0.080	3.5	57.6
	PE-NMe2(18:1(9Z)/20:4(5Z,8Z,11Z,14Z))	$M+NH_4$	$C_{45}H_{80}NO_8P$	30.7	0.080	3.5	57.6
	PE-NMe2(18:1(11Z)/20:4(5Z,8Z,11Z,14Z))	$M+NH_4$	$C_{45}H_{80}NO_8P$	30.7	0.080	3.5	57.6
	PE(20:3(5Z,8Z,11Z)/20:2(11Z,14Z))	$M+NH_4$	$C_{45}H_{80}NO_8P$	30.7	0.079	3.5	57.6
	PE(20:2(11Z,14Z)/20:3(5Z,8Z,11Z))	$M+NH_4$	$C_{45}H_{80}NO_8P$	30.7	0.079	3.5	57.6
	PE-NMe2(18:2(9Z,12Z)/20:3(5Z,8Z,11Z))	$M+NH_4$	$C_{45}H_{80}NO_8P$	30.7	0.079	3.5	57.6
	PE-NMe2(20:3(5Z,8Z,11Z)/18:2(9Z,12Z))	$M+NH_4$	$C_{45}H_{80}NO_8P$	30.7	0.079	3.5	57.6
	PE(20:3(8Z,11Z,14Z)/20:2(11Z,14Z))	$M+NH_4$	$C_{45}H_{80}NO_8P$	30.7	0.063	3.5	57.6
	PE(20:2(11Z,14Z)/20:3(8Z,11Z,14Z))	$M+NH_4$	$C_{45}H_{80}NO_8P$	30.7	0.063	3.5	57.6
	PE-NMe2(18:2(9Z,12Z)/20:3(8Z,11Z,14Z))	$M+NH_4$	$C_{45}H_{80}NO_8P$	30.7	0.063	3.5	57.6
	PE-NMe2(20:3(8Z,11Z,14Z)/18:2(9Z,12Z))	$M+NH_4$	$C_{45}H_{80}NO_8P$	30.7	0.063	3.5	57.6
	PE(18:4(6Z,9Z,12Z,15Z)/22:1(13Z))	$M+NH_4$	$C_{45}H_{80}NO_8P$	30.7	0.017	3.5	57.6
	PE(22:1(13Z)/18:4(6Z,9Z,12Z,15Z))	$M+NH_4$	$C_{45}H_{80}NO_8P$	30.7	0.017	3.5	57.6
	PE-NMe2(18:4(6Z,9Z,12Z,15Z)/20:1(11Z))	$M+NH_4$	$C_{45}H_{80}NO_8P$	30.7	0.017	3.5	57.6
	PE-NMe2(20:1(11Z)/18:4(6Z,9Z,12Z,15Z))	$M+NH_4$	$C_{45}H_{80}NO_8P$	30.7	0.017	3.5	57.6
	PE(20:5(5Z,8Z,11Z,14Z,17Z)/20:0)	$M+NH_4$	$C_{45}H_{80}NO_8P$	30.7	0	3.5	57.6
	PE(22:5(4Z,7Z,10Z,13Z,16Z)/18:0)	$M+NH_4$	$C_{45}H_{80}NO_8P$	30.7	0	3.5	57.6
	PE(22:5(7Z,10Z,13Z,16Z,19Z)/18:0)	$M+NH_4$	$C_{45}H_{80}NO_8P$	30.7	0	3.5	57.6
	PE-NMe2(20:5(5Z,8Z,11Z,14Z,17Z)/18:0)	$M+NH_4$	$C_{45}H_{80}NO_8P$	30.7	0	3.5	57.6
	PE-NMe2(22:5(4Z,7Z,10Z,13Z,16Z)/16:0)	$M+NH_4$	$C_{45}H_{80}NO_8P$	30.7	0	3.5	57.6
	PC(22:5(4Z,7Z,10Z,13Z,16Z)/15:0)	$M+NH_4$	$C_{45}H_{80}NO_8P$	30.7	0	3.5	57.6
	PC(22:5(7Z,10Z,13Z,16Z,19Z)/15:0)	$M+NH_4$	$C_{45}H_{80}NO_8P$	30.7	0	3.5	57.6
	PE-NMe2(22:5(7Z,10Z,13Z,16Z,19Z)/16:0)	$M+NH_4$	$C_{45}H_{80}NO_8P$	30.7	0	3.5	57.6
	PE(18:3(9Z,12Z,15Z)/22:2(13Z,16Z))	$M+NH_4$	$C_{45}H_{80}NO_8P$	30.7	0	3.5	57.6
	PE-NMe2(18:3(6Z,9Z,12Z)/20:2(11Z,14Z))	$M+NH_4$	$C_{45}H_{80}NO_8P$	30.7	0	3.5	57.6
	PE(18:3(6Z,9Z,12Z)/22:2(13Z,16Z))	$M+NH_4$	$C_{45}H_{80}NO_8P$	30.7	0	3.5	57.6
	PE-NMe2(18:3(9Z,12Z,15Z)/20:2(11Z,14Z))	$M+NH_4$	$C_{45}H_{80}NO_8P$	30.7	0	3.5	57.6
	PE(22:2(13Z,16Z)/18:3(6Z,9Z,12Z))	$M+NH_4$	$C_{45}H_{80}NO_8P$	30.7	0	3.5	57.6
	PE(22:2(13Z,16Z)/18:3(9Z,12Z,15Z))	$M+NH_4$	$C_{45}H_{80}NO_8P$	30.7	0	3.5	57.6
	PE(18:0/22:5(7Z,10Z,13Z,16Z,19Z))	$M+NH_4$	$C_{45}H_{80}NO_8P$	30.7	0	3.5	57.6
	PE-NMe2(20:2(11Z,14Z)/18:3(6Z,9Z,12Z))	$M+NH_4$	$C_{45}H_{80}NO_8P$	30.7	0	3.5	57.6

	PE-NMe2(20:2(11Z,14Z)/18:3(9Z,12Z,15Z))	M+NH4	$C_{45}H_{80}NO_8P$	30.7	0	3.5	57.6
	PE-NMe2(18:0/20:5(5Z,8Z,11Z,14Z,17Z))	M+NH4	$C_{45}H_{80}NO_8P$	30.7	0	3.5	57.6
	PE-NMe2(16:0/22:5(7Z,10Z,13Z,16Z,19Z))	M+NH4	$C_{45}H_{80}NO_8P$	30.7	0	3.5	57.6
	PC(15:0/22:5(4Z,7Z,10Z,13Z,16Z))	M+NH4	$C_{45}H_{80}NO_8P$	30.7	0	3.5	57.6
	PC(15:0/22:5(7Z,10Z,13Z,16Z,19Z))	M+NH4	$C_{45}H_{80}NO_8P$	30.7	0	3.5	57.6
	PE-NMe2(16:0/22:5(4Z,7Z,10Z,13Z,16Z))	M+NH4	$C_{45}H_{80}NO_8P$	30.7	0	3.5	57.6
	PE(18:0/22:5(4Z,7Z,10Z,13Z,16Z))	M+NH4	$C_{45}H_{80}NO_8P$	30.7	0	3.5	57.6
	PE(20:0/20:5(5Z,8Z,11Z,14Z,17Z))	M+NH4	$C_{45}H_{80}NO_8P$	30.7	0	3.5	57.6
	GPEtn(20:0/20:5)	M+NH4	$C_{45}H_{80}NO_8P$	30.7	0	3.5	57.6
	GPCho(15:1/22:4)	M+NH4	$C_{45}H_{80}NO_8P$	30.7	0	3.5	57.6
	GPEtn(24:4/16:1)	M+NH4	$C_{45}H_{80}NO_8P$	30.7	0	3.5	57.6
	GPCho(17:0/20:5)	M+NH4	$C_{45}H_{80}NO_8P$	30.7	0	3.5	57.6
	GPEtn(22:5/18:0)	M+NH4	$C_{45}H_{80}NO_8P$	30.7	0	3.5	57.6
	GPCho(17:1/20:4)	M+NH4	$C_{45}H_{80}NO_8P$	30.7	0	3.5	57.6
	GPCho(17:2/20:3)	M+NH4	$C_{45}H_{80}NO_8P$	30.7	0	3.5	57.6
	GPCho(20:3/17:2)	M+NH4	$C_{45}H_{80}NO_8P$	30.7	0	3.5	57.6
	GPCho(20:4/17:1)	M+NH4	$C_{45}H_{80}NO_8P$	30.7	0	3.5	57.6
	GPCho(20:5/17:0)	M+NH4	$C_{45}H_{80}NO_8P$	30.7	0	3.5	57.6
	GPEtn(22:4/18:1)	M+NH4	$C_{45}H_{80}NO_8P$	30.7	0	3.5	57.6
	GPEtn(22:2/18:3)	M+NH4	$C_{45}H_{80}NO_8P$	30.7	0	3.5	57.6
	GPEtn(22:1/18:4)	M+NH4	$C_{45}H_{80}NO_8P$	30.7	0	3.5	57.6
	GPEtn(20:5/20:0)	M+NH4	$C_{45}H_{80}NO_8P$	30.7	0	3.5	57.6
	GPEtn(20:4/20:1)	M+NH4	$C_{45}H_{80}NO_8P$	30.7	0	3.5	57.6
	GPEtn(20:3/20:2)	M+NH4	$C_{45}H_{80}NO_8P$	30.7	0	3.5	57.6
	GPEtn(20:2/20:3)	M+NH4	$C_{45}H_{80}NO_8P$	30.7	0	3.5	57.6
	GPCho(22:4/15:1)	M+NH4	$C_{45}H_{80}NO_8P$	30.7	0	3.5	57.6
	GPCho(22:5/15:0)	M+NH4	$C_{45}H_{80}NO_8P$	30.7	0	3.5	57.6
	GPEtn(16:1/24:4)	M+NH4	$C_{45}H_{80}NO_8P$	30.7	0	3.5	57.6
	GPEtn(18:0/22:5)	M+NH4	$C_{45}H_{80}NO_8P$	30.7	0	3.5	57.6
	GPEtn(18:1/22:4)	M+NH4	$C_{45}H_{80}NO_8P$	30.7	0	3.5	57.6
	GPEtn(20:1/20:4)	M+NH4	$C_{45}H_{80}NO_8P$	30.7	0	3.5	57.6
	GPEtn(18:3/22:2)	M+NH4	$C_{45}H_{80}NO_8P$	30.7	0	3.5	57.6
	GPEtn(18:4/22:1)	M+NH4	$C_{45}H_{80}NO_8P$	30.7	0	3.5	57.6
	GPCho(15:0/22:5)	M+NH4	$C_{45}H_{80}NO_8P$	30.7	0	3.5	57.6
B	7,13-Eperudien-15-oic acid	M-H	$C_{20}H_{32}O_2$	41.2	12.1	-3.4	97.7
	Copalic acid	M-H	$C_{20}H_{32}O_2$	41.2	12.1	-3.4	97.7
	Eicosatetraenoic acid	M-H	$C_{20}H_{32}O_2$	41.2	12.1	-3.4	97.7
	5,8,11,14-Icosatetraenoic Acid	M-H	$C_{20}H_{32}O_2$	41.2	12.1	-3.4	97.7
	Arachidonic acid	M-H	$C_{20}H_{32}O_2$	41.2	12.1	-3.4	97.7
	Cis-8,11,14,17-Eicosatetraenoic acid	M-H	$C_{20}H_{32}O_2$	41.2	12.1	-3.4	97.7
	2,6,10,14-Hexadecatetraenoic acid, 3,7,11,15-tetramethyl-, (Z,E,E)-	M-H	$C_{20}H_{32}O_2$	41.2	12.1	-3.4	97.7
	Drostanolone	M-H	$C_{20}H_{32}O_2$	41.0	11.5	-3.4	97.7
	Mesterolone	M-H	$C_{20}H_{32}O_2$	41.0	11.5	-3.4	97.7
	Mestaline	M-H	$C_{20}H_{32}O_2$	41.0	11.5	-3.4	97.7
	Methandriol	M-H	$C_{20}H_{32}O_2$	38.8	0.44	-3.4	97.7
	Oryzalexin E	M-H	$C_{20}H_{32}O_2$	38.8	0.43	-3.4	97.7
	ent-17-Hydroxy-16beta-kauran-19-al	M-H	$C_{20}H_{32}O_2$	38.7	0	-3.4	97.7
	Oryzalexin S	M-H	$C_{20}H_{32}O_2$	38.7	0	-3.4	97.7
	Yucalexin P21	M-H	$C_{20}H_{32}O_2$	38.7	0	-3.4	97.7
	Junicedral	M-H	$C_{20}H_{32}O_2$	38.7	0	-3.4	97.7
	Oryzalexin F	M-H	$C_{20}H_{32}O_2$	38.7	0	-3.4	97.7
	Oryzalexin D	M-H	$C_{20}H_{32}O_2$	38.7	0	-3.4	97.7
	Sideridiol	M-H	$C_{20}H_{32}O_2$	38.7	0	-3.4	97.7
	abietadiene-diol	M-H	$C_{20}H_{32}O_2$	38.7	0	-3.4	97.7
C	PS(6 keto-PGF1alpha/20:1(11Z))	M-H	$C_{46}H_{82}NO_{14}P$	35.9	3.0	0.48	77.2
	PS(20:1(11Z)/6 keto-PGF1alpha)	M-H	$C_{46}H_{82}NO_{14}P$	35.9	3.0	0.48	77.2

PS(TXB2/20:1(11Z))	M-H	$C_{46}H_{82}NO_{14}P$	35.9	3.0	0.48	77.2
PS(20:1(11Z)/TXB2)	M-H	$C_{46}H_{82}NO_{14}P$	35.9	3.0	0.48	77.2

The compound with the highest score value was accepted as CYP2C19 biomarker candidate when multiple compounds were identified.

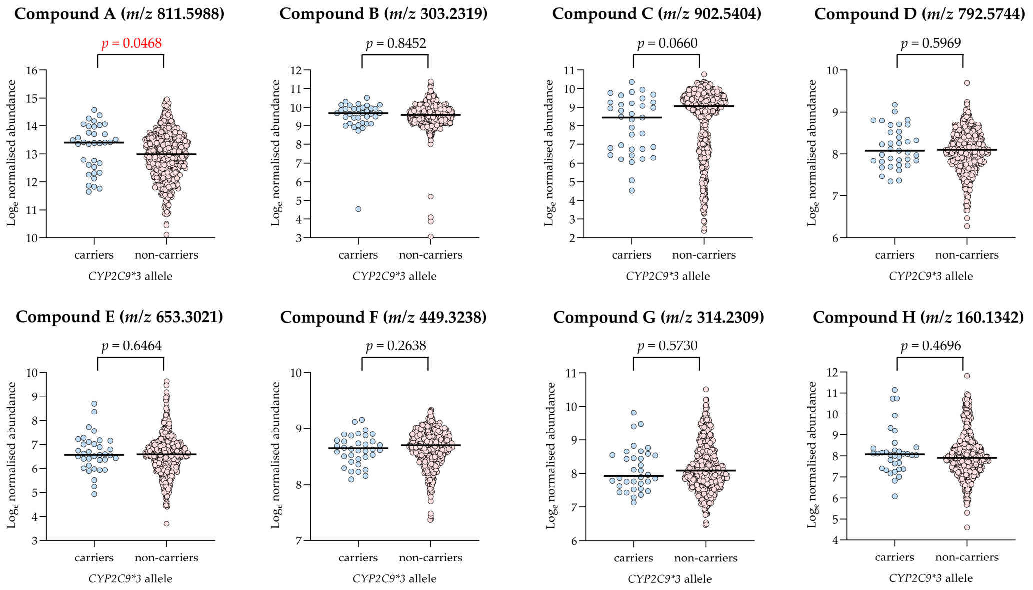

Figure S2. Comparison of log$_e$ normalized abundance of Compounds A–H between *CYP2C9*3* allele carriers and non-carriers. Data normality was analyzed by Shapiro-Wilk test. Statistical analyses were performed using Welch's *t*-test or Mann–Whitney *U* test. The horizontal line represents mean or median value. A p-value less than 0.05 was considered statistically significant.

Table S7. Optimal cutoff values, sensitivity, specificity in each receiver operating characteristic analysis.

Model	Cutoff value	Sensitivity	Specificity
Base	0.42	0.20	0.90
Compound A (*m/z* 811.5988)	0.37	0.58	0.58
Compound B (*m/z* 303.2319)	0.40	0.42	0.76
Compound C (*m/z* 902.5404)	0.35	0.64	0.52
Compound D (*m/z* 792.5744)	0.33	0.78	0.36
Compound E (*m/z* 653.3021)	0.41	0.34	0.84
Compound F (*m/z* 449.3238)	0.37	0.56	0.60
Compound G (*m/z* 314.2309)	0.39	0.55	0.64
Compound H (*m/z* 160.1342)	0.37	0.59	0.61

Only participants with C-reactive protein levels available were included in receiver operating characteristic analysis (extensive metabolizer, n = 230; poor metabolizer, n = 137). The base model included age, alanine aminotransaminase, C-reactive protein, estimated glomerular filtration rate, and cohort as covariates to estimate the probability of being a PM. Each candidate compound model was constructed by adding the corresponding $\log_e$ normalized abundance of candidate compound to the base model.
